# Protective effects of anandamide against cisplatin-induced peripheral neuropathy in rats

**DOI:** 10.3906/sag-2101-224

**Published:** 2021-06-12

**Authors:** Çiğdem ÇENGELLİ ÜNEL, Başak AYAZ, Şule AYDIN, Emel ULUPINAR, Orhan ÖZATİK, Bilgin KAYGISIZ, Engin YILDIRIM, Kevser EROL

**Affiliations:** 1 Department of Pharmacology, Faculty of Medicine, Eskişehir Osmangazi University, Eskişehir, Turkey; 2 Department of Interdisciplinary Neuroscience, Faculty of Medicine, Eskişehir Osmangazi University, Eskişehir, Turkey; 3 Department of Histology and Embryology, Faculty of Medicine, Kütahya Health Science University, Kütahya, Turkey; 4 Department of Pharmacology, Faculty of Medicine, Bahçeşehir University, İstanbul, Turkey

**Keywords:** Cis-diamminedichloroplatinum(II), n-arachidonoylethanolamide, ng nitroarginine methyl ester, nitric oxide, peripheral neuropathy

## Abstract

**Background/aim:**

Cisplatin (CIS) is an effective antineoplastic agent used in the treatment of several cancer types. Peripheral neuropathy is a major dose-limiting side-effect in CIS therapy. Cannabinoids may alleviate this painful side effect. This study investigated the analgesic effects of anandamide (AN) on CIS-induced peripheral neuropathy, in vitro effects of AN in CIS neurotoxicity, and the contribution of nitric oxide (NO) in this effect.

**Materials and methods:**

This is an experimental animal study. Primary dorsal root ganglion (DRG) cultures were prepared from one-day-old rats for in vitro investigations. DRG cells were incubated with CIS (100–300 μM), and AN (10, 50, 100, and 500 μM) was administered with the submaximal concentration of CIS. Female Sprague Dawley rats were divided into control, CIS, CIS+AN, CIS+AN+ L-NG-nitro arginine methyl ester (LNAME). CIS was administered 3 mg/kg i.p once weekly for 5 weeks. AN (1 mg/kg i.p) or in combination with 10 mg/kg i.p LNAME was administrated 30 min before CIS injection. Mechanical allodynia, thermal hyperalgesia, and tail clip tests were performed. After intracardiac perfusion, sciatic nerves (SN), and DRGs were isolated and semi-thin sections were stained with toluidine blue and investigated histologically. SPSS v. 21.0 and Sigma STAT 3.5 were used for statistical analysis. One/two way ANOVA, Kruskal–Wallis, and Wilcoxon signed ranks tests were used. A p-value of 0.05 was accepted as significant.

**Results:**

CIS caused significant mechanical allodynia. AN and AN+LNAME significantly increased hind paw withdrawal latency in mechanical allodynia test. The degenerated axons significantly increased in CIS group, while decreased in AN group. The frequency of larger neurons seemed to be higher in CIS+AN group.

**Conclusion:**

AN may be a therapeutic alternative for the treatment of CIS-induced peripheral neuropathy. However, its central adverse effects must be considered.

## 1. Introduction

Cisplatin (CIS) is a well-known chemotherapeutic agent used to treat a wide variety of tumors. Most of the patients under CIS therapy experience peripheral neuropathy which might cause dose limitation, discontinuation of therapy and decrease patients’ quality of life [1]. Painful neuropathy may initiate in several weeks of treatment and continue to several months after discontinuation of the therapy [2]. There are some underlying mechanisms suggested for the development of CIS-induced neuropathic pain. These are summarized as follows: mitochondrial dysfunction can arise due swollen and vacuolated mitochondria in axons and also by the release of intracellular calcium [3]; upregulation of some TRP channels in dorsal root ganglion (DRG) neurons can lead to hyper-responsiveness of nociceptors [4]; mitogen-activated protein kinase can be affected by the activation of p38 and ERK1/2 in DRG neurons along with down regulation of JNK/Sapk [5]. CIS can also activate NMDA receptors [6] that leads to increase in neuropeptide Y and substance P along with the alteration of calcitonin gene related peptide and somatostatin in DRG neurons [7]. However, the exact mechanism of CIS-induced peripheral neuropathy has not been fully elucidated. CIS has been found at higher levels in DRG neurons and causes detrimental effects which lead to neuronal dysfunction and cell death. This may cause irreversible structural and functional abnormalities in the peripheral nervous system in the long term [8]. Many agents have been proposed to manage chemotherapy-induced peripheral neuropathy. However, none of these agents has been proven effective.

The endocannabinoid system is one of the endogenous systems that is critical in the control and modulation of pain [9]. Cannabinoid (CB) receptors, CB1 and CB2 are promising therapeutic targets for the treatment of pain. CB receptor agonists have been shown to have antinociceptive effects in several neuropathic pain models [10]. Anandamide (AN), an endogenous cannabinoid, has been shown to cause antinociception in various experimental pain studies including neuropathic, inflammatory, and tumor pain and also generate full cannabinoid tetrad effects [11,12]. The tetrad model is used to detect potential effects of agents on CB1 receptors. After the acute systemic administration of CB1 agonist molecules, four characteristic effects are observed such as hypolocomotion, hypothermia, catalepsy, and analgesia [13]. Various mechanisms underlying the analgesic and neuroprotective effects of cannabinoids were suggested. Cannabinoids were reported to attenuate pain and to reduce oxidative stress via CB1 receptors [14]. In addition, CBs were shown to have neuroprotective effects [15] by antagonizing NMDA receptors [16]. AN was also shown to control pain modulation by acting at TRPV1 receptors [17]. Most of DRG neurons were shown to express mRNA for CB1 and these receptors were shown to co-localize with TRPV1 in some of small-diameter DRG neurons [18]. AN was identified as an endogenous ligand for TRPV1 receptors and high concentrations of AN activated them [19]. Besides CB1 receptor activation, their antiinflammatory NMDA antagonist effects and actions on TRPV1 receptors may contribute to the neuroprotective effects of AN.

Nitric oxide (NO) is a widespread signaling molecule that has a complex and diverse role in the modulation of pain [20]. Studies suggest that the expressions of NO synthase isoforms (NOS1 and NOS2) have been up-regulated in the spinal cord and DRG after nerve injury in animal models of neuropathic pain [21,22]. Additionally, NO synthase inhibitors, LNAME and 7-nitroindazole have been shown to alleviate acute or chronic pain [22]. Evidence also suggest that AN interacts with NO [23].

The aims of this study were to investigate: (i) the effects of AN on CIS-induced neurotoxicity of primary DRG neurons, (ii) the influences of AN on allodynia and hyperalgesia in CIS-induced peripheral neuropathy in rats and (iii) possible ability of nonselective NOS inhibitor lNAME to potentiate the effect of AN.

## 2. Materials and methods

### 2.1. DRG isolation and cell culture

The experimental procedures were approved by the Local Ethical Committee of Eskisehir Osmangazi University for the care and use of experimental animals (permit number: 362/2013). The primary cultures of DRG were prepared as previously described [24]. Rats were purchased from Medical and Surgical Research Center of Eskisehir Osmangazi University. Briefly, DRGs were collected from 1-day-old Sprague Dawley rats and kept in ice-cold, sterile calcium- and magnesium-free modified Hank’s balanced salt solution (HBSS) (Sigma Aldrich; Lonza, Belgium). Then, DRG neurons were incubated with trypsin solution (0.25% trypsin–0.02% EDTA) (Gibco, Thermo Fisher Scientific, Waltham, MA, USA) at 37 °C for 10 min. Cells were dissociated by trituration with a fire-polished Pasteur pipette and plated in poly-D-lysine (Sigma Aldrich, St. Louis, MO, USA)-coated culture plates. Dulbecco’s modified Eagle’s medium (DMEM) was used as culture media and changed twice a week.

DRG neurons became ready to be used in neurotoxicity experiments after in vitro incubation period of 8–10 days. DRG (approximately 5 × 10^3^ cells/well) were incubated overnight and left to adhere onto surface of coated 96-well culture plates in drug-free DMEM medium. Then, CIS was added to the wells with gradually increasing concentrations as applied in our previous study (100, 200, 300 μM) [25] and the cells were incubated for 24 h with the drug. The neurotoxic effects of CIS were evaluated by incubating the cells with CIS alone or in combination of submaximal concentration of CIS (200 μM) and AN (10, 50, 100, and 500 μM). The viability of cultured DRG cells was detected by using MTT (3-(4,5-dimethylthiazolyl-2)-2,5-diphenyltetrazolium bromide) assay method [25]. Absorbance was measured at 540 nm with a microplate reader (Multiscan EX; Franklin, MA, USA).

### 2.2. CIS-induced peripheral neuropathy and behavioral studies

The study is an experimental animal study. The experiments were performed according to principles of the Local Ethical Committee of Eskisehir Osmangazi University for the care and use of experimental animals (protocol number: 394-2/2016). Twenty-four, female adult Sprague Dawley rats (160–220 g, n = 6/group) were used and peripheral neuropathy was induced by once a week intraperitoneal (ip) injection of 3 mg/kg CIS (50 mg/100 mL concentrated solution for intravenous infusion, Koçak Farma, Tekirdag, Turkey) for 5 weeks as indicated in a previous study [26]. Group size of n *=* 6 animals for behavioral experiments was determined by sample size estimation using G*Power (v3.1) [27] to detect size effect in a post hoc test with type 1 and 2 error rates of 5 and 20%, respectively. Rats were kept under conditions of a light–dark (12/12) cycle and free access to food and water ad libitum for 1 week to habituate and divided into the following groups:

- **Control (vehicle) group:** 2 mL intraperitoneal (i.p) saline was injected once a week for 5 weeks.
- **CIS-induced neuropathic pain group:** 3 mg/kg i.p CIS was injected once a week for 5 weeks.
- **AN- and CIS-administrated group:** 1 mg/kg i.p AN (Sigma) and 3 mg/kg i.p CIS were injected once a week for 5 weeks.
- **AN-, LNAME-, and CIS-administrated group**: 1 mg/kg i.p AN, 10 mg/kg LNAME (Sigma), and 3 mg/kg i.p CIS were injected once a week for 5 weeks.

All treatments were administered at 9:00 a.m., and CIS was administered 30 min after injections. AN and LNAME were dissolved in saline. LNAME was administered immediately after AN injection. Saline (2 mL) was also given to prevent CIS-induced nephrotoxicity. Mechanical, thermal, and tail withdrawal latencies were assessed on day 0 (baseline) and on the 6th day after each drug injections. Drug administrations were ended at 35th day. After 2 weeks which was needed for the structural changes, animals were euthanized to collect tissues for the measurement of pathological parameters [28].

#### 2.2.1. Mechanical allodynia test

The mechanical allodynia was evaluated as previously described [24]. Testing was performed once a week during 5 weeks between 9.00 a.m. and 12:00 p.m. All animals were first tested for baseline measurement before the drug administrations. Rats were placed inside acrylic cages with a perforated metal platform and were left to habituate for approximately 30 min before the measurement. Briefly, mechanical allodynia of right hind paw was assessed by using dynamic plantar test (Ugo Basile S.R.L. 37400-002, Italy). Mechanic stimuli was delivered to the plantar surface with an increasing force (0–50 g in 20 s) using a 0.5 mm diameter filament until the animal twitches its paw. Consecutive 3–5 measurements were recorded with at least 5 min intervals for each rat. Cut off force was accepted 50 g to avoid tissue damage.

#### 2.2.2. Thermal hyperalgesia test

The thermal sensitivity was evaluated as previously described [24]. Testing was performed once a week for 5 weeks between 9.00 a.m. and 12:00 p.m. All animals were first tested before the drug administrations. Rats were placed inside acrylic cages with a transparent glass floor and were left to habituate for approximately 30 min before the measurement. Briefly, heat sensitivity of right hind paw was assessed by using thermal hyperalgesia test apparatus (Ugo Basile S.R.L. 37370-002, Italy). Thermal stimuli were delivered to the plantar surface of the hind paw with a targeted beam of radiant heat. Consecutive 3–5 measurements were conducted for each rat at least 5 min intervals. Cut off time was accepted as 20 s to avoid tissue damage.

#### 2.2.3. Tail clip test

Central spinal antinociception was assessed as previously described on the tail of rats by using artery clip [24]. Artery clip was clamped down 1 cm above from the end of the tail. The time spent for biting or turning to tail was recorded as seconds. Cut-off time was accepted as 20 s.

#### 2.2.4. Cannabinoid tetrad

The cannabinoid tetrad was studied in only AN administrated neuropathy group. According to cannabinoid tetrad model; we evaluated their hypothermia, catalepsy, analgesia and locomotion [29]. Analgesic effects were assessed using a thermal hyperalgesia test as described above. In bar test, a wooden bar 9 cm above from the ground was used for catalepsy. Forepaws of animals were placed over the bar and the time spent on the bar recorded as seconds. Hypothermia of animals were measured by digital thermometer which was inserted into the animals’ rectum. Locomotor activity test was evaluated by using activity meter (MAY Commat, Ankara, Turkey). Total movement of each animal was recorded for 5 min. All of the cannabinoid tetrad tests were applied before drug administrations and 1 h after the last AN administration.

### 2.3. Analysis of DRG and sciatic nerves

At the 8th week, intra-cardiac perfusion was performed using 4% sodium phosphate buffer (pH 7.4) under ketamine-xylazine anesthesia (ketamine 80 mg/kg, xylazine 12 mg/kg) for morphological evaluations. SN and associated DRG were dissected and removed in the same fixative solution. DRG were collected and embedded in epon-araldite resin. DRG sections of 700 nm thickness were cut from 3 depths of the samples by microtome and stained with toluidine blue. The sections were observed under light microscope (Olympus BX5; Tokyo, Japan). Soma areas of DRG were calculated using Image J analysis program.

Sciatic nerves (SN) (1 cm) were cut proximal to the trifurcation and fixed with 2.5% glutaraldehyde solution in 0.1 M phosphate buffer. After 24 h of fixation at 4 °C, samples of the nerve segments were rinsed with phosphate buffer and post fixed with 1% osmium tetroxide in 0.1 M phosphate buffer for 2 h at room temperature. Then samples were dehydrated in graded solutions of ethanol and embedded in epon resin. SN sections (700 nm thick) were stained with toluidine blue and observed with light microscope (Olympus BX5; Tokyo, Japan). Degenerated axons were designated according to two criteria including myelin debris formation and finer degeneration in axons. The number of normal and degenerated axonal fibers was counted and the ratio of degenerated/normal (deg/nor) was calculated [30].

### 2.4. Statistical analysis

SPSS 21.0 (IBM, USA) and Sigma STAT 3.5 (Systat Software Inc, USA) were used for the statistical analysis. Data that were not normally distributed were represented as mean ± SEM. Values of p < 0.05 was accepted as significant. One-way ANOVA and Tukey tests were used for the evaluation of CIS neurotoxicity in vitro and Kruskal–Wallis test was used for the evaluation of AN effects in vitro. The two-way ANOVA for repeated measures and Tukey test for multiple comparisons were used in the assessment of statistical analysis for behavioral studies (mechanical allodynia, thermal hyperalgesia, and tail clip). Wilcoxon signed ranks test was for the analysis of cannabinoid tetrad. Student–Newman–Keuls method was used in the multiple comparisons of size-frequency histogram of DRG neurons.

## 3. Results

### 3.1. Neurotoxicity experiments

**Figure 1:**
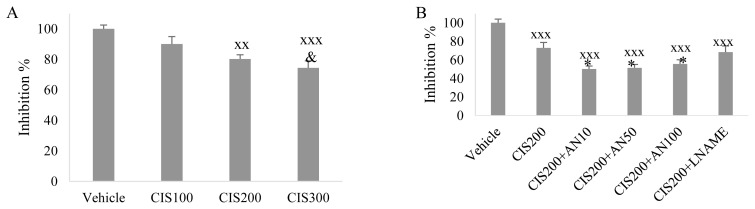
Inhibition percentage values were obtained from MTT assay (A) the concentration-dependent inhibitory effects of Cisplatin (CIS) (100–300 μM); (B) The effects of CIS (200 μM) alone and combination of CIS (200 μM) and Anandamide (AN) (10, 50, 100; AN10, AN50, AN100). (xx: p < 0.01, xxx: p < 0.001 vs. Vehicle; &: p < 0.05 vs. CIS 100 μM; *: p < 0.05 vs. CIS 200 μM, n = 10). Bars represent mean ± SEM.

CIS administration induced a concentration dependent neurotoxicity on DRG neurons and CIS 200 μM was detected as the concentration which caused minimum neurotoxic effect on these cells (Figure 1a, p < 0.001). To assess in vitro effects of AN, different concentrations of AN (10, 50, and 100 μM) were combined with CIS 200 μM. AN 10, 50 and 100 μM (Figure 1b, p < 0.001) caused neurotoxicity compared to control which were significantly higher than CIS 200 μM itself (Figure 1b, p = 0.013, p = 0.014, p = 0.037 respectively). LNAME did not cause any difference in cisplatin neurotoxicity (Figure 1b, p < 0.001).

### 3.2. Behavioral studies

#### 3.2.1. Mechanical allodynia

**Figure 2:**
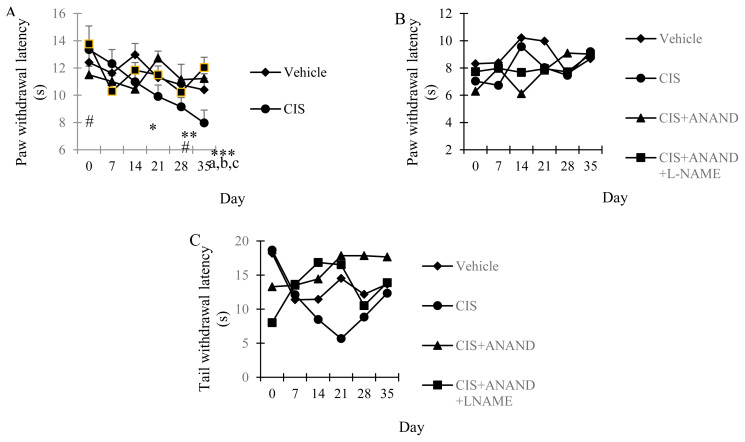
Paw withdrawal latencies (A) in mechanical allodynia test. ($: p < 0.05 vs Vehicle (control) group on the day 35; *:p < 0.05; **:p < 0.01 Cisplatin (CIS) vs. baseline; #: p < 0.05; Cisplatin+Anandamide+LNAME (CIS+AN+LNAME) vs. baseline; a: p < 0.05 CIS vs. Vehicle, b: p < 0.01 CIS+AN vs CIS and c: p < 0.01 CIS+AN+LNAME vs. CIS group on the 35th day). (B) in thermal hyperalgesia test (C) Tail withdrawal latencies in tail clip test by days. Basal measurement indicated day 0 (no injection received). Bars represent mean ± SEM.

There was no significant difference in the baseline values (day 0) of paw withdrawal latencies among all groups. In addition, there was also no significance between all measurements of control group. CIS administration significantly decreased the paw withdrawal latency of rats compared to control (p = 0.003) and also baseline which was beginning from the 21nd day (p = 0.003) of administration and continued to decrease in 28th and 35th days (Figure 2a, p = 0.016, p < 0.001 respectively). Concurrent administration of AN (p < 0.01) or AN+LNAME (p < 0.001) with CIS treatment significantly increased the paw withdrawal latencies compared to control after 35 days of drug injections (Figure 2a).

### 3.3. Thermal hyperalgesia

CIS and concurrent administration of AN or AN+LNAME with CIS treatment didn’t cause any significant change in the paw withdrawal latencies of rats (Figure 2b).

### 3.4. Tail clip

No significant difference was detected in the tail withdrawal latencies of control, CIS, CIS+AN, and CIS+AN+LNAME groups (Figure 2c).

### 3.5. Cannabinoid tetrad

**Figure 3:**
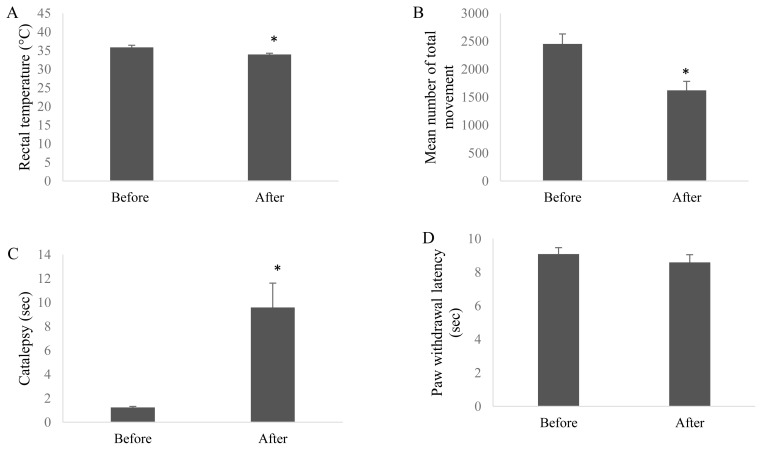
Acute central effects of systematically administered Anandamide (AN) by testing cannabinoid tetrad. The tests were used to investigate the central effects of AN. (A) rectal temperature (for hypothermia); (B) spontaneous locomotor activity (for hypolocomotion); (C) bar test (for catalepsy); (D) plantar test (for analgesia). Tests were performed before and 1 h after AN administration. (Bars represent mean ± SEM. *: p < 0.05; **: p < 0.01 with respect to value received before AN treatment.). Bars represent mean ± SEM.

AN administration significantly decreased the rectal temperature (Figure 3a, p = 0.028), significantly reduced the total movement (Figure 3b, p = 0.046) and significantly prolonged the catalepsy time (Figure 3c, p = 0.028) of rats compared to pre-administration. There was no significant alteration in the paw withdrawal latencies of rats in thermal hyperalgesia test after AN administration (Figure 3d).

### 3.6. Dorsal root ganglia and sciatic nerves

**Figure 4:**
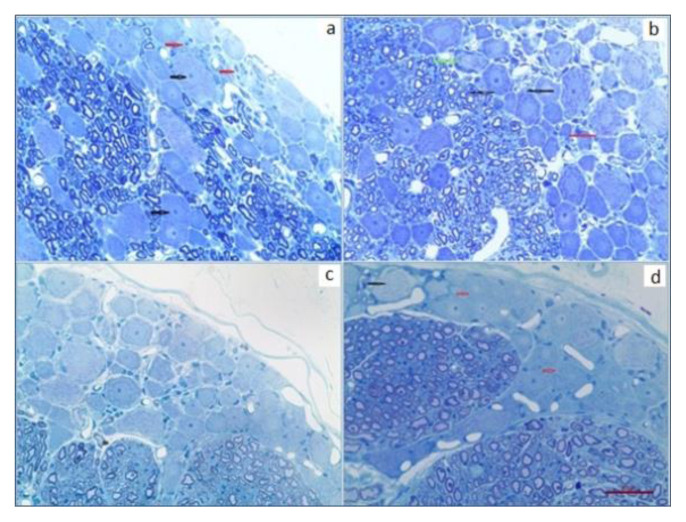
Semi-thin sections of DRG neurons. (a) Control (vehicle), (b) CIS, (c) AN, (d) CIS+AN+LNAME groups (scale represents 50 μm.)

Morphological examinations of DRG neurons showed that nuclei were centrally located (black arrows) and there were satellite cells around ganglion cells (red arrows) in control group (Figure 4a). In CIS group, microvacuolization was seen in ganglion cells (black arrows) and membrane lines of cells were lost (red arrow). Some of the ganglion cells were also swelled (green arrow), (Figure 4b). In CIS+AN group, there were less microvacuolization ganglion cells and membrane lines were clear compared to CIS group. Swelling of cytoplasm also was not observed in this group (Figure 4c). In CIS+AN+LNAME group, vacuolization was observed in ganglion cells (black arrow). Membrane lines were also lost (red arrow). Injury of DRG cells was clearly observed (Figure 4d).

**Figure 5:**
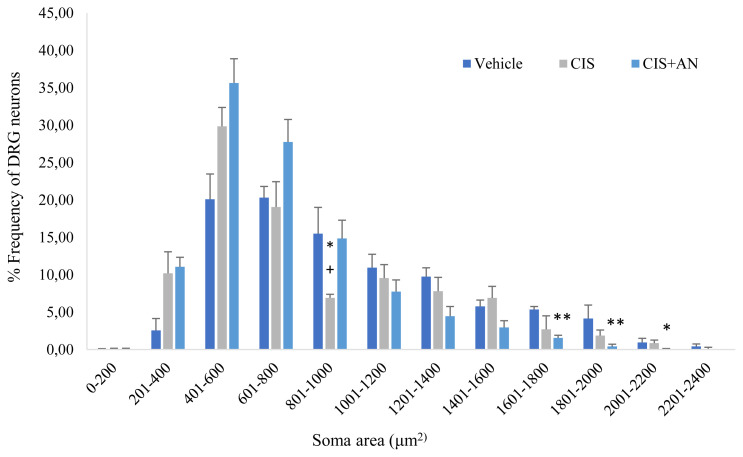
Histogram of cross-sectional areas of dorsal root ganglion (DRG) neurons. Bars represent mean ± SEM. *:p < 0.05, **:p < 0.01 vs. Vehicle; +: p < 0.05 vs. Cisplatin+Anandamide (CIS+AN).

Cross sectional soma areas of DRG neurons in each group were analyzed. Frequency distribution histogram of soma areas showed that frequency of DRG neurons with small soma areas was higher in CIS than in control. In CIS treated rats, the frequency of DRG neurons decreased when soma areas increased. Especially the frequency of DRG neurons between 801–1000 μm^2^ significantly decreased by CIS treatment and increased by the AN treatment (Figure 5, p = 0.028).

**Figure 6:**
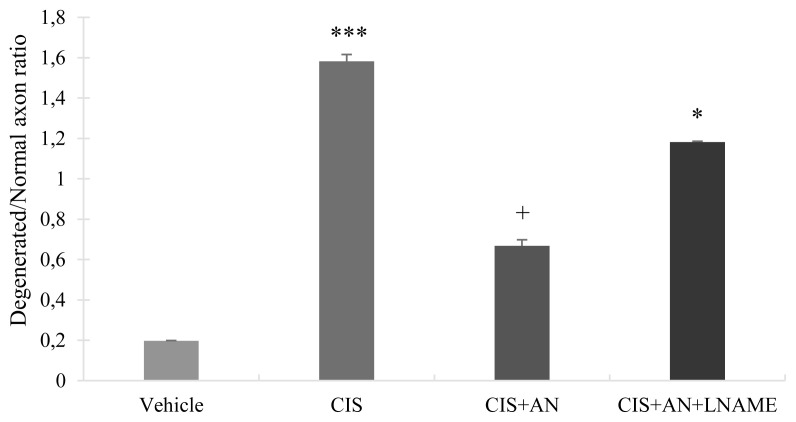
The ratio of degenerated/normal axon in sciatic nerve (SN) (*: p < 0.05; ***: p < 0.001 vs. Vehicle; +: vs. Cisplatin (CIS). Bars represent mean ± SEM. (CIS+AN: Cisplatin+Anandamide, CIS+AN+LNAME: Cisplatin+Anandamide+LNAME)

The Deg/Nor axon ratio in SN was significantly increased in CIS (p < 0.001) and CIS+AN+LNAME (p = 0.02) groups but not in CIS+AN group compared to control. Concurrent administration of AN with CIS treatment significantly reduced the Deg/Nor axon ratio compared to CIS group (p = 0.04). The difference is more in CIS+AN group than in CIS+AN-LNAME group compared to CIS group (Figure 6).

**Figure 7:**
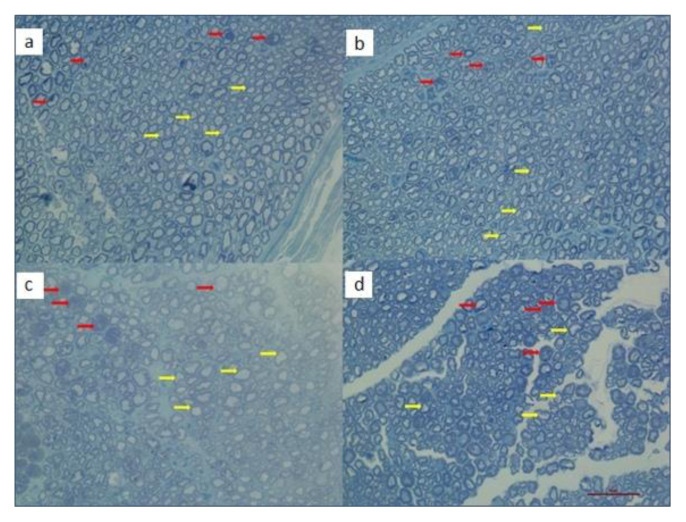
Semi-thin sections of sciatic nerve (SN). (a) Control (Vehicle), (b) CIS, (c) CIS+AN, (d) CIS+AN+LNAME groups. Red arrows indicate degenerated myelinated fibers and yellow arrows indicate normal and regenerated myelinated fibers (scale represents 50 μm).

Morphological examinations of SN showed that degeneration in myelinated fibers were more in CIS group compared to control group (Figures 7a and b). The degeneration in CIS+AN group were lower than in CIS group but higher than in control (Figure 7c). Degenerated myelinated fibers in CIS+AN+LNAME group were higher than control and CIS+AN groups (Figure 7d).

## 4. Discussion

Cannabinoids are targeted molecules for the treatment of a series of diseases including neuropathic pain. In our study CIS induced peripheral neuropathy in vivo and neurotoxicity in vitro. Chronically administrated cannabinoid AN was able to counteract the inhibitory effects of cisplatin in mechanical allodynia test and the same result was obtained if AN plus LNAME were given. In addition, higher concentrations of AN ameliorated the structural abnormalities induced by CIS. The histological alterations induced by CIS in DRG cells and SN were also improved by AN but that the additional presence of LNAME attenuates this effect. AN was also effective in three paradigms of cannabinoid tetrad.

The basic mechanism of CIS neurotoxicity involves DRG damage. For the first time we investigated the potential protective effects of AN in primary culture of DRG cells. The permeability of vascularization and lacking of blood-brain barrier in DRG neurons can lead to free passage, accumulation and toxicity of chemicals in these cells [31]. Thus, DRG neurons are defenseless against toxic effects of CIS. In our study, CIS induced concentration-dependent neurotoxicity in DRG cell culture (Figure 1a) as shown before [32]. Low concentrations (10, 50, 100 μM) of AN significantly increased the toxicity of CIS. However, high concentration (500 μM) of AN seemed to dampen the neurotoxic effects of CIS which needs further investigations (Figure 1b). In the second part of the study, the effects of AN were investigated in CIS-induced peripheral neuropathy in vivo. CIS produced mechanical allodynia that was manifested by 21st day and was maintained until the end of the experiment (35th day). Hyperalgesia or hypoalgesia to heat was notably absent. Besides, no significant alteration was detected in tail clip test (Figure 2). Similar result was demonstrated in a previous study [33]. On the 35th day of our study in which the neuropathy was so significant, AN and CIS+AN+LNAME groups increased the paw withdrawal latency in mechanical allodynia test. However, the combination with LNAME did not induce any significant change from the effects of AN alone in neuropathic rats. (Figure 2a).

Tail clip to investigate nociception at the spinal level and thermal hyperalgesia test at supraspinal level were also used in our study as indicated earlier [34]. According to our results any significant change was not observed in these tests. In a previous study AN was reported to reduce thermal hypersensitivity in partial SN ligation neuropathic pain model [35]. Intraplantar injection of AN was also demonstrated to inhibit thermal hyperalgesia induced by carrageenan [36]. The inconsistent results of the study may be due to different thermal hypersensitivity of animals in various neuropathic pain models.

In nociception NO has dual effects and it may induce either pro-algesia or analgesia [37]. The underlying mechanism of these effects involve NMDA receptors and COX enzymes for hyperalgesic action and cGMP-PKG-ATP sensitive potassium channels pathway for the analgesic effects of NO [37]. The maintenance of neuropathic pain behaviors was reported to be modulated by the production of NO [38]. NOS inhibitors were reported to promote antinociception at various levels of sensory system and in different experimental models [39,40]. However, in our study NOS inhibition did not alter the actions of AN. This may be because of unstable nature of this enzyme. Even if NOS was reported to have pronociceptive effects in neuropathic pain, different experimental models of neuropathic pain (such as transaction, crush, hypoxia or ligation) may lead to contradictory results. NO pathway also keeps interaction with other transmitter pathways. Especially NO-cGMP-PKG pathway plays a critical role in peripheral antinociception induced by cannabinoids [41]. Cannabinoids were shown to have potent analgesic effects in different experimental models of neuropathic pain [42,43]. In presence of peripheral nerve injury, significant alterations were reported in CB receptor binding. It was reported that receptor binding was upregulated by surgery in wild type animals, however there was no alteration in NOS knockout animals [44]. Thus under pathological conditions such as inflammation or pain, the level of cannabinoid binding and interactions between NO-cGMP pathway and cannabinoid system were altered [44]. These interactions may affect the nociceptive behaviors measured in different experimental models of neuropathic pain.

Cannabinoids are newer suggested agents in the management of pain. However, their undesirable central adverse effects such as dizziness, dysphoria, euphoria, ‘feeling high’ and sedation seemed to limit their clinical use [45]. Especially CB1 agonists crossing blood-brain barrier may cause these central effects. To check the central effects of AN, cannabinoid tetrad was assessed. AN (1 mg/kg) at the end of 35th day significantly induced most of the cannabinoid tetrad effects including hypothermia, hypomobility, and catalepsy with respect to pretreatment results (Figure 3). Low doses of cannabinoids were known to promote both of depressant and stimulatory effects but higher doses may cause central depression like effects [46]. Interestingly in our study, chronically applied 1 mg/kg AN induced the signs of cannabinoid tetrad. This antiallodynic dose of AN unfortunately caused central psychoactive effects. The difference could be caused by the differences between pathophysiology of animal models of neuropathic pain or the experimental protocols used.

In histological evaluations, structural abnormalities observed in CIS group were ameliorated by AN (Figures 4b and c). Furthermore, a significant decrease in frequency of DRG neurons corresponding to CIS treated rats was found in our study especially having soma areas between 801–1000 μm^2^ (p = 0.038). In addition, the number of DRG neurons having smaller soma areas were observed to be high in CIS treated rats. This may be because of the fact that CIS caused to atrophy in neuron parts including cell body, nucleus and nucleolus [47]. Our results were also consistent with the results of other studies confirming that the number of DRG neurons was also reduced with the CIS treatment [47,48]. Moreover, AN caused an increase in the frequency of DRG neurons having soma areas between 801–1000 μm^2^ (p = 0.038) with respect to CIS treated group. DRG neurons with diameters >35 μm (large neurons) were shown to express high levels of GPR55, while those with diameters <35 μm (small ones) do not [49]. Therefore, we also noticed that CIS reduced the frequency of DRG neurons at 801–1000 μm^2^ and AN reversed its activity (Figure 5). AN was suggested to increase intracellular calcium by activating these receptors. In the histological examination of semi-thin sections of DRG revealed severe pathology induced by CIS. Microvacuolizations inside ganglion cells were observed and cell membranes were lost in CIS treated rats. This pathology was ameliorated by the addition of AN to CIS; however, in CIS+ AN+LNAME group, the similar injury was observed in CIS group (Figure 6). This kind of effects of AN may be related to its activation of CB1 receptors and IP3 signaling pathway and stimulating the release of Ca^2+^ from intracellular stores [16].

SN morphometry was correlated with the morphometric analysis of DRG neurons. Both the deg/nor axon ratio and degeneration of myelinated fibers were significantly lower in AN and CIS+AN+LNAME groups than that of CIS group. However, degeneration was more in LNAME combination group when compared to AN group (Figure 7). It seems that AN might have a potential role to restore structural abnormalities induced by CIS. In addition, according to our results LNAME was able to worsen the structural effects of AN.

## 5. Conclusion

Consequently, based on our results AN and its combination with LNAME were able to prevent mechanical allodynia induced by CIS. In addition, AN alone could also alleviate the toxic effects of CIS and ameliorate the structural abnormalities of DRG cells and SN induced by CIS treatment. AN could have been an alternative for the treatment of peripheral neuropathy in cancer patients receiving CIS therapy. However, 1 mg/kg chronically applied AN was shown to cause central effects in our study. NOS inhibitor LNAME did not change the palliative effects of AN in mechanical allodynia, on the other hand it worsened the structural pathology of DRG neurons and SN. Another mechanism rather than NOS inhibition seems to play a role in the obvious effects of AN. LNAME and AN may interact in another pathway causing a decrease in the effects of AN structurally. Further studies are needed to clarify the exact mechanism behind neuroprotective and antiallodynic effects of AN.
